# Online Structural-Health Monitoring of Glass Fiber-Reinforced Thermoplastics Using Different Carbon Allotropes in the Interphase

**DOI:** 10.3390/ma11071075

**Published:** 2018-06-25

**Authors:** Michael Thomas Müller, Hendrik Florian Pötzsch, Uwe Gohs, Gert Heinrich

**Affiliations:** 1 Leibniz Institute of Polymer Research Dresden, Hohe Str. 6, D-01069 Dresden, Germany; hendrik_florian.poetzsch@tu-dresden.de (H.F.P.); gohs@ipfdd.de (U.G.); gheinrich@ipfdd.de (G.H.); 2 Institute of Textile Machinery and High Performance Material Technology, Technische Universität Dresden, D-01062 Dresden, Germany

**Keywords:** glass fiber, interphase, in-situ sensor, glass fiber-reinforced thermoplastics, carbon filler

## Abstract

An electromechanical response behavior is realized by nanostructuring the glass fiber interphase with different highly electrically conductive carbon allotropes like carbon nanotubes (CNT), graphene nanoplatelets (GNP), or conductive carbon black (CB). The operational capability of these multifunctional glass fibers for an online structural-health monitoring is demonstrated in endless glass fiber-reinforced polypropylene. The electromechanical response behavior, during a static or dynamic three-point bending test of various carbon modifications, shows qualitative differences in the signal quality and sensitivity due to the different aspect ratios of the nanoparticles and the associated electrically conductive network densities in the interphase. Depending on the embedding position within the glass fiber-reinforced composite compression, shear and tension loadings of the fibers can be distinguished by different characteristics of the corresponding electrical signal. The occurrence of irreversible signal changes during the dynamic loading can be attributed to filler reorientation processes caused by polymer creeping or by destruction of electrically conductive paths by cracks in the glass fiber interphase.

## 1. Introduction

Lightweight constructions gain increasing acceptance and will partially replace metals on the market in the long term. Glass fiber-(GF) reinforced thermoplastics feature good characteristics in terms of stiffness, strength, lightweight and chemical resistance combined with design flexibility. To overcome the barriers to entry into the mass market, the long-term stability of lightweight constructions has to be ensured. Consequently, detailed information on the inner bulk phase and the GF interphase are required in order to get basic knowledge about the failure pattern of GF-reinforced thermoplastics. Integrated structural-health sensors are able to deliver the desired information. However, mostly the reinforcing fibers were substituted by sensor fibers e.g., carbon, piezo-resistive ceramics, or Bragg gratings [1,2,3,4,5,6,7,8,9]. Unfortunately, no information of the most vital region [10,11,12,13] for the stress transfer between GF and the surrounding matrix, e.g., polypropylene (PP), is accessible with such systems. For the investigation of the mechanical stress level in the GF thermoplastic interphase, an embedding of an electrically conductive network allows the detection of mechanical stress-strain behavior by monitoring the response of the electrically conductive network, due to the piezo-resistive effect [14,15,16,17,18,19,20]. Wiegand et al. [15] showed that the electrical resistance change of carbon nanotubes nanostructured GF is suitable to measure the interphase deformation in a quantitative manner. Furthermore, it was proved that the early stage damage can be predicted by on-line resistance measurements since the GF interphase fails prior to ultimate structural failure. 

In this study, different carbon allotropes compared to carbon nanotubes (CNT), e.g., carbon black (CB) and graphite nanoplatelets (GNP), were tested regarding the implementability for structural-health monitoring. Therefore, the carbon fillers were dispersed into the sizing which is applied during the production of GF by a roller coating process. Based on the induced electrical percolated filler network in the GF interphase, a piezo-resistive response can be detected during applied mechanical loadings. Within this approach, the composite itself becomes a sensor as individual nanostructured GF are placed in mechanical critical regions. Thus, we produced endless GF-reinforced PP composites with embedded nanostructured GF to distinguish between different signal characteristics of compression, shear and tension loadings. The nanostructuring was realized with different filler shapes (1D to 3D [21]), such as fiber-like and sheet-like as well as spherical.

## 2. Experimental

### 2.1. Materials and Processing

#### Glass Fiber Spinning and Interface Modification

**Figure 1:**
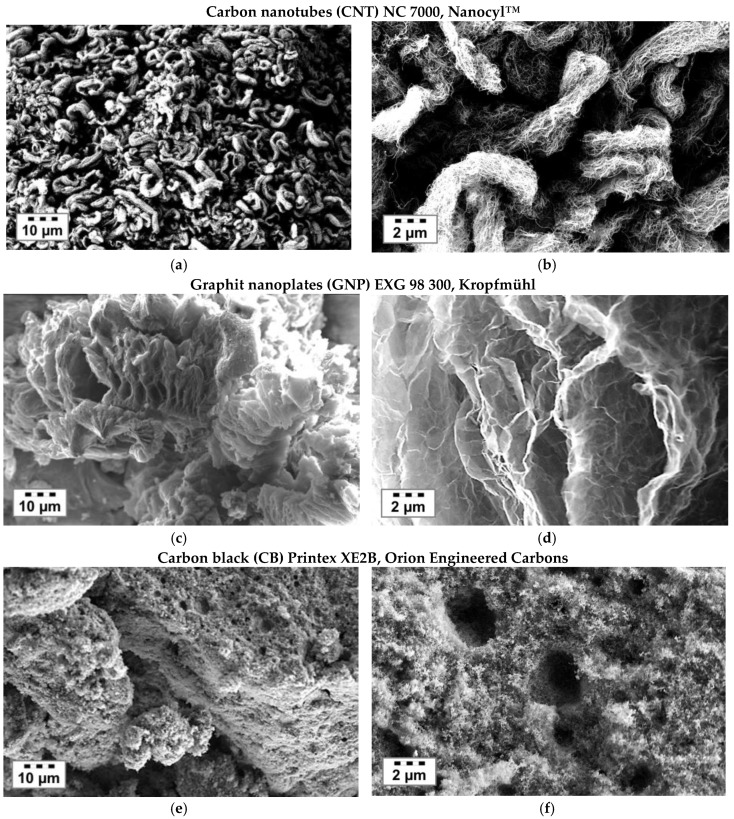
SEM images of different as-received commercially available carbon filler. (**a**,**b**) Multiwall Carbon nanotubes (NC 7000, Nanocyl™); (**c**,**d**) Graphit nanoplates (EXG 98 300, Kropfmühl); (**e**,**f**) Carbon black (Printex XE2B, Orion Engineered Carbons).

Two different types of yarns were melt-spun. At first, an commingled hybrid yarn [22,23], 50/50 vol. % consisting of continuous E-glass filaments (average diameter 17 µm) and PP filaments (average diameter 22 µm, PP: HG455FB, Borealis, Vienna, Austria) were simultaneously spun and sized using an aqueous 3-aminopropyltriethoxysilane (Dynasylan^®^ AMEO, Evonik, Essen, Germany, 1 wt. %) and maleic grafted PP film former (10 wt. %, Aquacer 598, Byk, Essen, Germany) solution. Further, an E-glass filament with an average diameter of 17 μm was melt spun and sized with an aqueous 1 wt. % 3-Aminopropyltriethoxysilan (AMEO) solution. Subsequently, these E-glass/AMEO filament yarns were coated with a carbon filler sizing using a continuous coating system type (Werner Mathis AG, Oberhasli, Switzerland). The chosen filler content within the sizing was for CNT 2.7 wt. % (Nanocyl™ NC7000, Sambreville, Belgium), GNP 4 wt. % (Graphit Kropfmühl, EXG 98 300, Hauzenberg, Germany) and for CB 9 wt. % (Printex XE2B, Orion Engineered Carbons, Senningerberg, Luxembourg), relative to the solid content of the film former. The different carbon fillers (Table 1, Figure 1) were dispersed into PP film former (20 wt. % Aquacer 598, Byk, Wesel, Germany) by an ultrasonic treatment process using a continuous flow cell (Hielscher UP200S + D14K, Teltow, Germany). The sonication cell is water chilled to dissipate the heat which was produced during ultrasonic treatment. 

**Figure 2:**
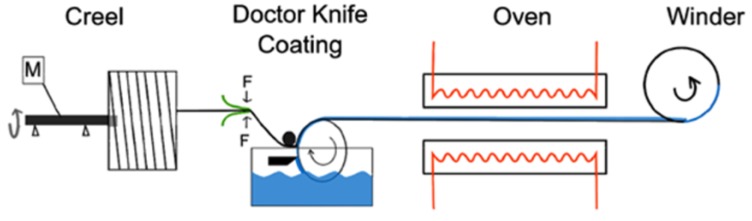
Schematic process of a secondary fiber coating device, consisting four individual devices [14].

The sensor fiber coating process consists of four individual stages (Figure 2). Firstly, the creel rotates the E-glass roving at a constant spin rate in order to prevent a yarn twist during the unwinding. Afterwards, a roller picks up the sizing from a bath while the thickness of the sizing film on the roller is controlled using a doctor blade (30 µm gap). The doctor blading fabricates a well-defined film thickness on the roller surface, which is controlled by the gap size between the blade and the roller surface. The glass fibers will be pulled through the sizing film, whereby the sizing is transferred from the roller to the glass fiber in a well-defined manner. Subsequently, the coated yarn passes through a drying process in the oven in order to evaporate the water of the sizing and to form a dried film on the GF yarn (drying temperature of 150 °C, with upstream infrared heating). Finally, the yarn is wound onto a bobbin at a velocity of 7.8 m/min. 

### 2.2. Filler Dispersion Characterization

To determine the state of filler macro-dispersion of the aqueous carbon filler dispersion, light transmission microscopy (LM) investigations were performed according to the standard ISO-18553 [29] on drop cast film between two glass sheets. To ensure an equal layer thickness, the applied contact pressure was largely kept constant. An Olympus microscope BH2 combined with a camera DP71 (Olympus Deutschland GmbH, Hamburg, Germany) was used. The area ratio of the carbon filler structures was determined from the LM images using the software Olympus Stream Image Analysis by calculating the ratio (A_r_ in %) of the area of remaining carbon filler structures (A_A_) to the total area of the image area (A_0_), as shown in Equation (1).

(1) $$A_{r}=\frac{A_{A}}{A_{0}}$$

whereby, according to the ISO-18553 standard [29], only structures over 19.2 μm^2^ were regarded. For its calculation, the optical gray value threshold was adjusted manually to detect the particles based on the contrast differences between the polymer matrix and remaining particle structures. For quantification, 10 films were investigated for each filler dispersion and the standard deviation as a measure of reproducibility is shown in the plots as coloured area. 

### 2.3. Filler Dispersion Characterization

The electrical surface resistivity of dried drop cast films was determined according to the standard ASTM-D257 [30] (ASTM International, West Conshohocken, PA, USA). At least three film samples were measured to obtain the arithmetic mean value with the associated standard deviation of resistivity. The measurements of the samples with resistances <10^7^ Ω were performed using a Loresta-GP electrometer in combination with an four-point ESP-probe (Mitsubishi Chemical Analytech Co. Ltd., Yamato, Japan, external source electrodes spacing 15 mm and measuring electrodes spacing 5 mm) and with resistances >10^7^ Ω were performed using a Hiresta-UP in combination with ring URS-probe (Mitsubishi Chemical Analytech Co. Ltd., Yamato, Japan, external circular electrode ø 17.8 mm, internal circular electrode ø 5.9 mm, measuring electrodes spacing 5.1 mm). At least 10 measurements per film were performed.

### 2.4. GF-Composite Preparation

**Figure 3:**
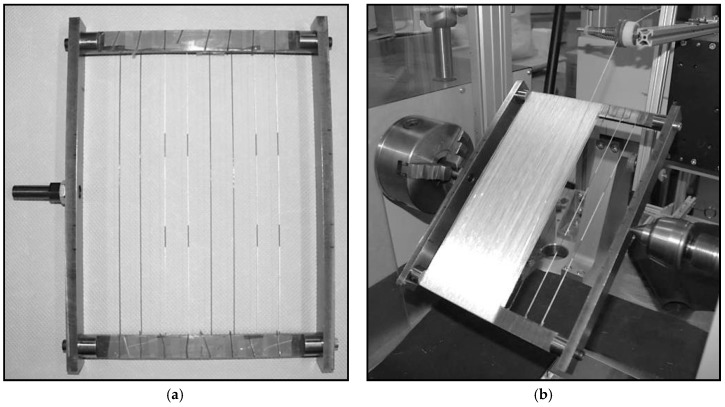
Sensor fiber placement in a rectangular mandrel (with and without silver paint pattern for improved conductivity) by adhesive tape (**a**) during the GF/PP unidirectional composites winding process (**b**).

The preparation of the unidirectional GF composites for the electromechanical response behavior testing is conducted by yarn winding process. As shown in Figure 3a, the nanostructured GF yarns were placed on a steel mandrel frame with and without using a conductive silver paint noticeable. Subsequently, five layers of nonconductive hybrid yarns were wound onto the frame in the direction of the carbon filler coated yarns (Figure 3b). Afterwards, the outer layer of the nanostructures GF yarns was fixed onto both sides using an adhesive tape, followed by an outer covering consisting of two further layers of nonconductive hybrid yarn. 

**Figure 4:**
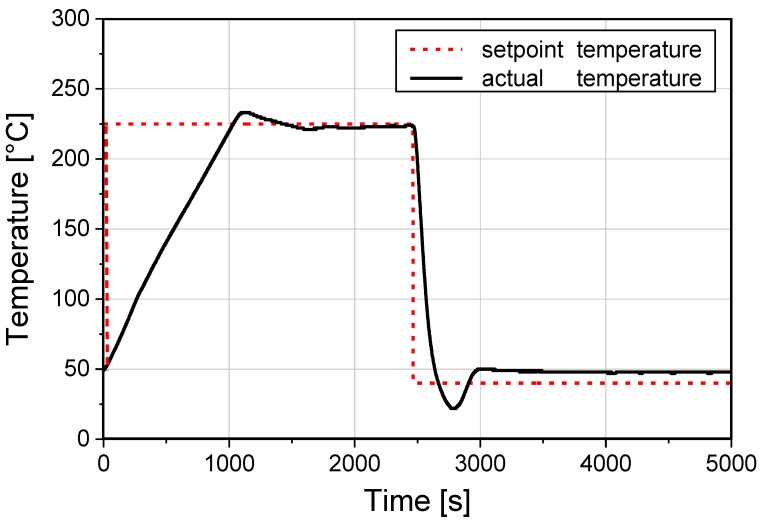
Set heating rate and the resulting actual temperature of the compression molding process.

The hybrid yarn was consolidated by compression moulding (K207, Rucks GmbH, Glauchau, Germany) at 225 °C. The consolidation procedure is shown in Figure 4. After the heating-up phase, the temperature of 225 °C (10 K/min) at 10 bar compression was kept constant for 21 min to ensure a full yarn melt-up [31]. Subsequently, the pressure was increased up to the point where the glass fibers were fully wetted (45 bar, 2 min), followed by cooling down (70 K/min) to room temperature. The test specimen were prepared using a diamond circular saw; all specimens were conducted with electrical cables using silver adhesive in order to facilitate electrical measurements.

### 2.5. Electro-Mechanical Response during 3-Point Bending Test

**Figure 5:**
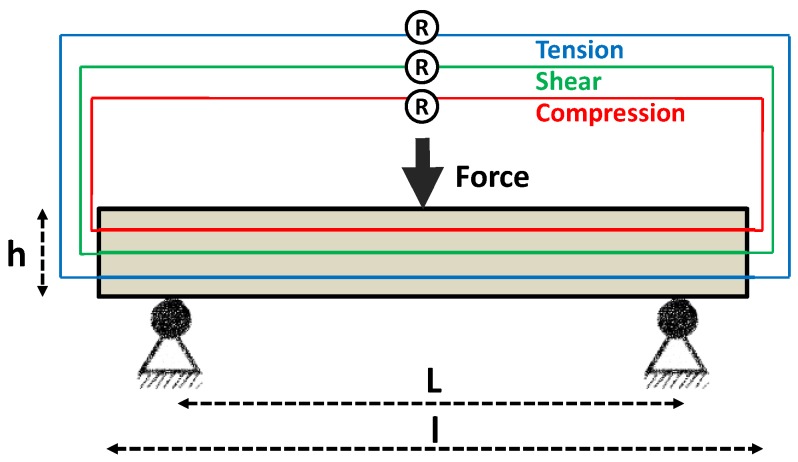
Test device, specimen and sensor layout (according to locus of maximum compression, maximum shear and maximum flexural stresses) for the three-point bending test on a rectangular unidirectional GF/PP composite with the direction of fibers along the specimen’s length axis [15].

In order to monitor the electromechanical response during three-point bending, the sensor fibers, each consisting of a bundle of 204 nanostructured glass filaments, were placed in the planes of maximum stresses. In total, three sensor fibers were embedded into each specimen according to detect the compression, shear, and flexural stresses (Figure 5). 

(2) $$G_{F}=\frac{\frac{\Delta R}{R}}{\frac{\Delta l}{l}}$$

The three-point bending test was performed in accordance to DIN EN 2562 [32] using rectangular specimens with a length (l) of 80 mm, a width (b) of 20 mm and a height (h) of 1.5 mm. The span length (L) was set to 50 mm. The electrical surface resistance change of the 3 sensor fibers was individually recorded using a Keithley 2000 electrometer. The data recording was realized by a LabView program on a personal computer. The sensitivity of the sensor fiber was determined by the gauge factor (G_F_), which is defined by Equation (2), the ratio of relative change in electrical surface resistance ($\frac{\Delta R}{R}$), to the mechanical strain ($\frac{\Delta l}{l}$) in the linear range (starting by first signal reply up to 1% deformation).

## 3. Results and Discussion

### 3.1. Filler Dispersion Preparation

Highly electrically conductive nanomaterials are needed in order to prepare GF with high electromechanical responsiveness by a continuous surface nanocoating. Therefore, we chose three different commercially available carbon nanofillers in accordance to their best intrinsic electrical conductivity values. To compare the electromechanical response behavior of different carbon modifications, such as fiber-like (carbon nanotubes), sheet-like (graphene) and spherical (carbon black) modifications (1D to 3D; [21]), were selected (Figure 1). For a homogeneous GF nanostructuring process, a stable CNT, GNP or CB dispersion with an excellent state of dispersion is of utmost importance. We used a commercially available aqueous PP emulsion, which is stabilized with nonionic additives. Due to the additive excess and the presence of PP micelles, the respective carbon fillers can be easily stabilized by an ultrasonic supported dispersion treatment.

**Figure 6:**
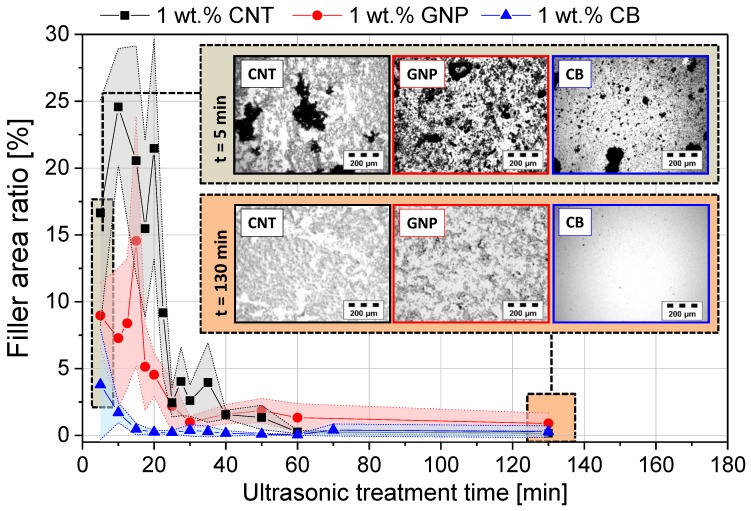
Development of the carbon filler macro-dispersion in aqueous PP emulsion tracked by optical light transmission microscopy using the primary filler area ratio.

Figure 6 shows the dispersion kinetic of the three different carbon fillers evaluated by the area ratio of visible filler agglomerates. All fillers followed the same dependency. With increasing ultrasonic treatment time, the state of the macro-dispersion is enhanced. This is reflected in the decrease of filler area ratio of primarily agglomerates. Whereby, the well-dispersed nanoparticles cannot be detected anymore due to the resolution limit of LM. It has to be taken into account that the values of initial area ratio are on different levels at the beginning of ultrasonic treatment. However, different treatment times for the respective filler are needed in order to achieve an excellent state of macro-dispersion. In the case of CB, a nearly agglomerate free dispersion was observed after 20 min. In contrast, CNT and GNP need much more time (up to 60 min) in order to reach a dispersed state. The interim increase of the primary area ratio during the first 20 min can be attributed to the filler agglomerate expansion to overcome the CNT entanglement and GNP stacking. In contrast, CB did not show such behavior due to its spherical morphology. In the respect to compare the influence of the different filler morphology on the electromechanical response behavior of nanostructured GF, an equivalent state of dispersion is required. Consequently, all used fiber sizings were prepared by an ultrasonic treatment of 60 min in order to ensure the same energy input.

### 3.2. Electrical Properties

**Figure 7:**
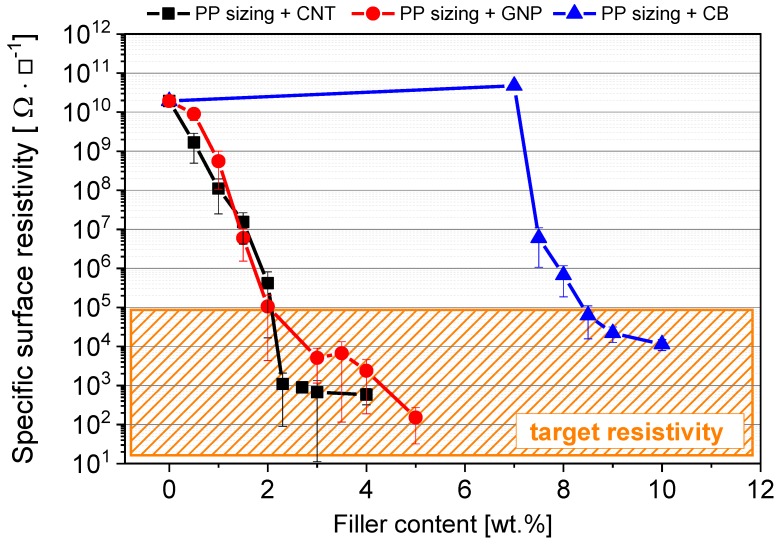
Electrical percolation behavior of different used carbon filler in polypropylene (PP), measured on consolidated drop-casted films on glass sheets.

Measuring the electrical surface resistivity of the different PP/filler dispersions, in order to optimize the required filler content for achieving a certain low electrical surface resistivity, is the next necessary stage for designing a electromechanical response ability. Figure 7 shows the electrical percolation behavior of the three different carbon filler modifications. The electrical percolation threshold for CNT and GNP material was obtained between 1 wt. % and 3 wt. %, for CB between 7 wt. % and 9 wt. %. A higher threshold for CB is reasonable, due to the spherical filler shape with associated low aspect ratio, whereby more particles are needed to build up an electrically percolated conductive network.

To realize a suitable electrical conductivity for our electromechanical response sensor, at least 2 wt. % of the CNT or GNP and 8.5 wt. % of the CB material is required. These filler contents are all near the electrical percolation threshold range which itself is very sensitive to processing conditions, whereby in the worst case the sensing ability getting lost. Therefore, we used fiber sizings with a filler content above the percolation threshold. Hence 2.7 wt. % of CNT, 4 wt. % GNP and 9 wt. % of CB were used to produce the sizings for the nanostructuring process.

### 3.3. Interphase Modification

For the preparation of an electrical conductive glass fiber interphase region, beside the electrically conductive filler network of the used sizing, a homogeneous coating on the glass fiber surface is necessary as well. Wiegand et al. [14] pointed out that the electrical conductivity depends significantly on the average sizing thickness. Consequently, a coating thickness of more than 450 nm is needed for sensing applications. This is the more than the double magnitude of a typical sizing thickness of 80–200 nm. Otherwise, the defects of coating increase and lead to a nonconductive interphase. Since the thickness of the final sizing film after coating and consolidation process depends mainly on the solid content of the applied sizing, a higher solid polymer content of 20 wt. % was used for the nanostructured sizings in this study. Other important parameters that influence the film formation of the sizing during the coating process are the fiber velocity and the temperature of the consolidation process. In this study, a temperature of 150 °C and a velocity of 7.8 m/min were used based on the postulated resistivity-velocity-temperature dependency of Wiegand et al. [14]. As a result, GF with an electrical resistivity of 7.3 × 10^4^ Ω/□ using CNT, of 1.2 × 10^6^ Ω/□ using GNP and of 1.7 × 10^5^ Ω/□ using CB were obtained and all resistivity values are suitable for the desired sensor application. 

### 3.4. Electro-Mechanical Response Behaviour

**Figure 8:**
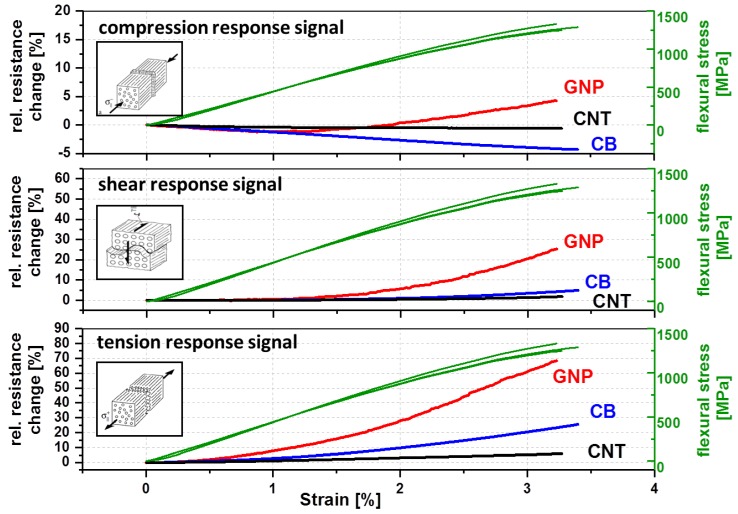
Electro-mechanical response behavior of nanostructured sensor fiber using different carbon filler (CNT, GNP, CB) embed in the planes of maximum stresses for compression, shear and tension, during a quasi-static three-point bending (Span to Length L/h ratio of 33).

For the determination of the strain-sensing behavior of the nanostructured GF in PP composites, a three-point bending test was chosen. In a static test assembly, the online resistance measurements show a clear correlation between the measured electrical signal and the applied mechanical deformation (Figure 8). Qualitative differences in the signal sensitivity and the deformation until the first signal reply are obtained when using different carbon allotropes (Table 2 and Figure 8). This behavior is based on the different aspect ratios and filler geometries with the associated densities of the electrically conductive networks. 

As expected, the first signal reply and sensor yarn response during mechanical loading depends on the embedding position. Most pronounced difference of the first signal reply values (Table 2 and Figure 8) between the carbon allotropes, were obtained by applied shear or tension stresses. Whereby, the deformation until the first signal replies in the case of compression stresses is low and practically identical for all fillers, since the electrically conductive network density per volume will increase for all filler-matrix systems. The CB filler in the interphase region required generally in all stress cases low filler displacements distances (0.5–0.17%) for changes in the electrically conductive network due to the very low aspect ratio. The displacements distances for CNT (0.63%) and GNP (0.48%) fillers by shear stress is relatively long, which is explainable by the higher aspect ratios. Hence, the filler particles are slipping against each other without losing electrical contact. Through the filler alignment of GNP platelets and the CNT fibers along the GF, lower displacements distances, which act transversal to the alignment, are necessary to enhance the resistivity for tension stresses compared to shear stresses.

An equal filler shape-stress mode dependency will be observed for sensitivity (gauge factor, Table 3) in the linear deformation range up to 1% strain and partial also in the afterwards non-linear deformation range, from 1% to the sample failure (Figure 8). Independently from the carbon allotrope, the highest resulting gauge factors (sensitivities) were obtained in the case of tension stress, the lowest factors were found for shear stress. The compression stress factors are in between. The reason for higher resistivity changes is the elongation of the interphase during tension stress, whereby the average sizing thickness decreases and the electron tunnel distances increase, since the electrically conductive filler network expands. Referring to the filler shape, the GNP material leads, compared to other fillers, to the highest sensitivity in all stress modes followed by carbon black. This is also reflected in the relative resistance change values of strain at the break point (Figure 8). The change of the electrical response signal of GNP (red line) is the highest in comparison to carbon black (blue line) and CNT (black line). 

**Figure 9:**
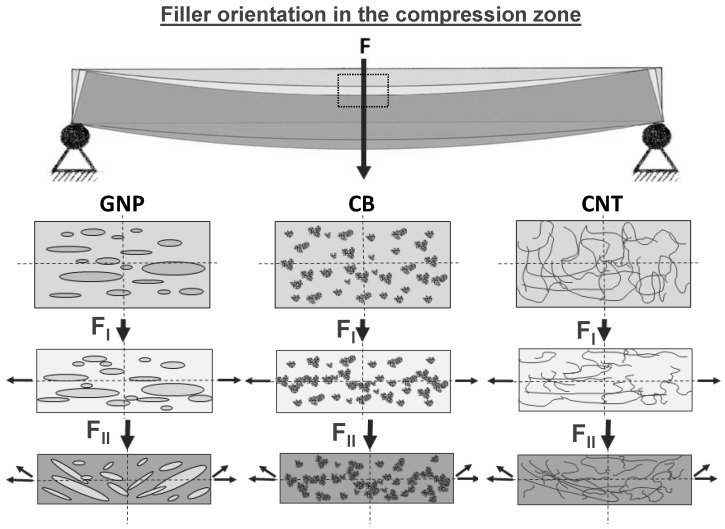
Orientation effects of different carbon allotropes on the nanostructured sensor fiber interphase during three-point bending test in the compression zone; exemplary shown for three different strain states.

**Figure 10:**
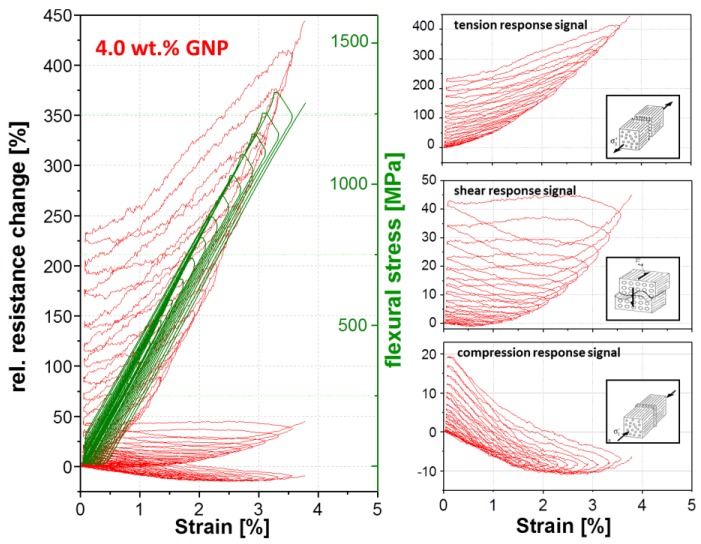
Cyclic electromechanical response behavior of nanostructured sensor fiber using 4 wt. % GNP embed in the planes of maximum stresses for compression, shear and tension, during a quasi-static three-point bending (Span to Length L/h ratio of 33, a stepwise increase of a mechanical loading of 50 MPa).

Except the compression stress, all used carbon allotropes show the similar signal characteristics during mechanical loading. During compression, the resistance of the used CNT and CB filler is not increased upon loading but tends to be decreasing until sample failure and remains below the unstrained value. The GNP shows a different signal dependence on strain during compression. At the beginning, the resistance decreased (strain ≤ 1.2%) due to the volume compression of the electrically conductive network, and at higher strain values the resistance increased. Such behavior could be caused by the platelets’ shape. The platelet size amount to serval microns tends to tilt at strain values above 1.2% during three-point bending (Figure 9). Therefore, the particle distance increases, whereby the resistance simultaneously increases. Such a signal characteristic can also be observed during the cyclic test were the mechanical load stepwise increases, whereby the tilt effect is increasing at each cycle (Figure 10). 

**Figure 11:**
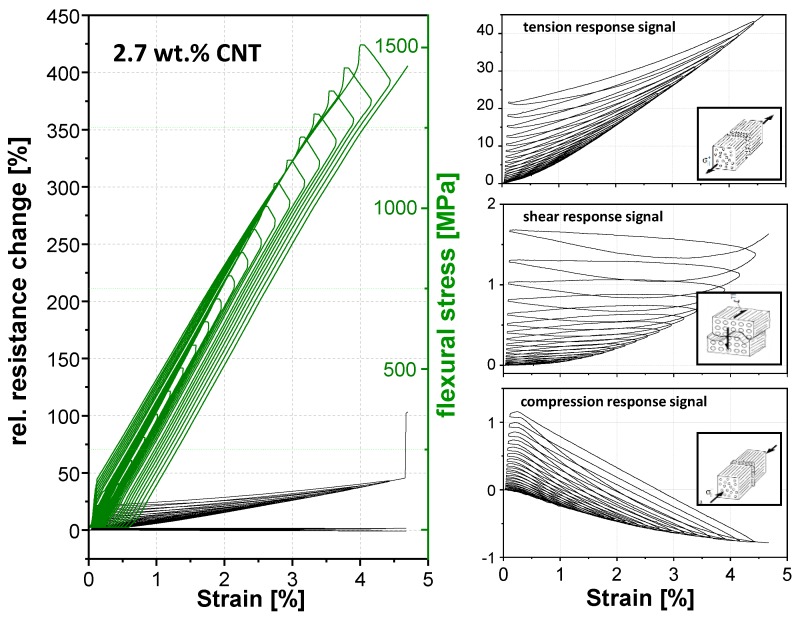
Cyclic electromechanical response behavior of nanostructured sensor fiber using 2.7 wt. % CNT embed in the planes of maximum stresses for compression, shear and tension, during a quasi-static three-point bending (Span to Length L/h ratio of 33, a stepwise increase of a mechanical loading of 50 MPa).

**Figure 12:**
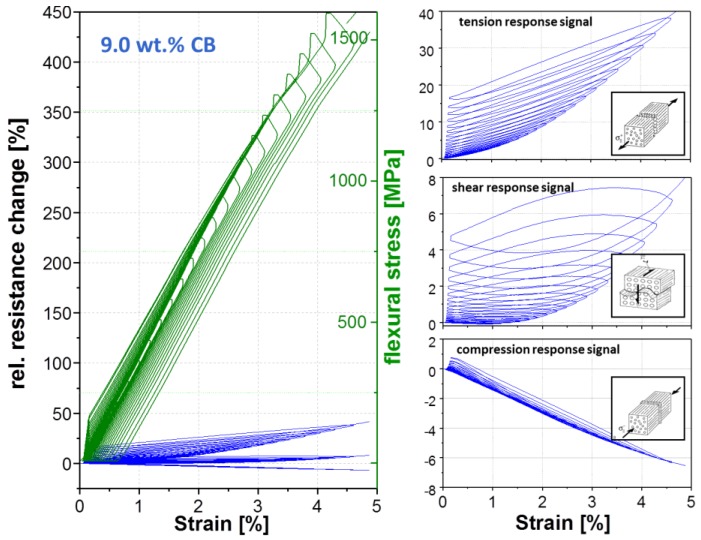
Cyclic electromechanical response behavior of nanostructured sensor fiber using 9 wt. % CB embed in the planes of maximum stresses for compression, shear and tension, during a quasi-static three-point bending (span to length L/h ratio of 33, a stepwise increase of a mechanical loading of 50 MPa).

A cyclic three-point bending test was performed in order to distinguish between reversible and irreversible effects on the sensor signal, depending on used carbon filler, by a stepwise increase of the mechanical load of 50 MPa. As expected, the sensitivity levels for the respective fillers is similar for the static test. The GNP material shows the highest sensitivity in this group and the CNT filler has the lowest one. Figure 10, Figure 11 and Figure 12 show that the mechanical loading and unloading hysteresis, is replied by a sensor signal hysteresis. The response upon mechanical loading corresponds nearly the quasi-static behavior. However, an irreversible change of resistance in the unstrained state occurs for all three fillers and stress modes during unloading. This irreversible part can be attributed to filler reorientation processes caused by polymer creeping and to a destruction of electrically conductive paths by cracks. During mechanical loading, the signal response of the tension mode shows only an irreversible resistance change. The electrical resistance increased by each mechanical loading cycle, which is an indication that cracks in the GF interphase region occur, which obviously propagate and further lead to further crack tip opening during the dynamic loading. Contrarily to this irreversible signal response, the compression and shear mode shows a combination of a reversible and irreversible process in the interphase. We assume that the cracks which are generated during the dynamic loading may again merged together leading to partly restoring of electrically conductive paths. Especially in the shear cycling, a certain resistance recovery to lower values is visible for all carbon allotropes during the cyclic mechanical loading.

## 4. Summary and Conclusions

Structural-health monitoring of GF-reinforced thermoplastics can be achieved by nanostructuring the GF interphase by using different highly electrically conductive carbon allotropes. The multifunctional interphases were achieved by applying a CNT, GNP, or CB modified sizing system onto GF prior to their consolidation. The electromechanical response behavior during a three-point bending test of various carbon modifications, fiber like (CNT), sheet-like (GNP) or spherical (CB) modifications, shows qualitative differences in the signal quality and sensitivity, due to the differing aspect ratios and the associated electrically conductive network densities. Static and cyclic tests show that the highest sensitivity (gauge factor) was obtained using sheet-like GNP material followed by the spherical CB filler. Further, the electromechanical response behavior during mechanical loading depends on the embedding position, whereby compression, shear, and tension loadings show different signal characteristics enabling a differentiation between these loadings. During cyclic mechanical loading, an irreversible resistance change in the unstrained state occurs. This irreversible part is independent of the filler shape and can be attributed to their reorientation caused by polymer creeping and to a destruction of electrically conductive paths by cracks. Such a hysteresis can be used to predict the composite failure. Therefore, additional long-time cyclic tests are required to acquire a comprehensive knowledge about the proposed correlation in order to determine the best matching type of carbon allotrope for on-line structural-health monitoring of GF-reinforced thermoplastics.

## Figures and Tables

**Table 1:** Specifications of the used commercial carbon filler material powders.

Material as Named by the Producer/Producer	Morphology	Average Particle Size d_50_ [µm]	Thickness/Diameter	Electrical Conductivity [S∙cm^−1^]	Specific Surface [m^2^∙g^−1^]	Bulk Density [kg∙m^−3^]
Nanocyl™ NC7000 (CNT) Nanocyl S.A.	fiber	>675 [24]	Ø 9.5 nm [25]	15 [26] ^*2^	250–300 [25]	66 [24]
EXG 98 300 (GNP) Graphit Kropfmühl	lamellar	305 ^*3^	-	3 [26] ^*2^	>300 [27]	1 ^*1^
Printex XE2B (CB) Orion Engineered Carbons	spherical	60 ^*3^	Ø 30–35 nm [28]	20 [26] ^*2^	1000 [28]	100–400 [28]

^*1^ In-house measurement according to EN 1097-3; ^*2^ at 30 MPa compression pressure; ^*3^ in-house laser diffraction measurement.

**Table 2:** Test specimen deformation until the first signal reply of nanostructured sensor fiber using different carbon fillers (CNT, GNP, CB) embedded in the planes of maximum stresses for compression, shear and tension, during a quasi-static three-point bending test.

Sample	Strain [%] Compression	Strain [%] Shear	Strain [%] Tension
CNT (2.7 wt. %)	0.07 ± 0.03	0.63 ± 0.1	0.07 ± 0.002
GNP (4 wt. %)	0.03 ± 0.02	0.48 ± 0.2	0.14 ± 0.06
CB (9 wt. %)	0.07 ± 0.02	0.17 ± 0.01	0.05 ± 0.01

**Table 3:** Sensitivity values (gauge factor) in a linear range (up to 1% deformation) of nanostructured sensor fiber using different carbon filler (CNT, GNP, CB) embed in the planes of maximum stresses for compression, shear and tension, during a quasi-static three-point bending test.

Sample	Gauge Factor Compression	Gauge Factor Shear	Gauge Factor Tension
CNT (2.7 wt. %)	−40 ± 1	20 ± 1	120 ± 2
GNP (4 wt. %)	−130 ± 2	120 ± 11	900 ± 18
CB (9 wt. %)	−130 ± 1	20 ± 1	290 ± 4
